# Nuclear receptor co-factor TBL1X/TBL1XR1 T cell activity protects against atherosclerosis

**DOI:** 10.1016/j.molmet.2026.102318

**Published:** 2026-01-13

**Authors:** Sahika Cingir Koker, Amit Mhamane, Julia Geppert, George Shakir, Raquel Guillamat-Prats, Bingni Chen, Pernilla Katra, Martina Geiger, Foivos-Filippos Tsokanos, Gretchen Wolff, Julia Szendrödi, Maria Rohm, Carolin Daniel, Lars Maegdefessel, Sabine Steffens, Stephan Herzig

**Affiliations:** 1 Institute for Diabetes and Cancer, Helmholtz Center Munich, Neuherberg, Germany; 2 German Center for Diabetes Research (DZD), Neuherberg, Germany; 3 Chair Molecular Metabolic Control, Technical University Munich, Munich, Germany; 4 Institute for Cardiovascular Prevention, Ludwig-Maximilians-Universität Munich, Germany; 5 DZHK (German Center for Cardiovascular Research), Partner Site Munich Heart Alliance, Germany; 6 Research Division Type 1 Diabetes Immunology, Helmholtz Diabetes Center at Helmholtz Zentrum München, 80939 Munich, Germany; 7 Division of Clinical Pharmacology, Department of Medicine IV, Ludwig-Maximilians-Universität München, Munich, Germany; 8 Department of Inner Medicine I, Heidelberg University Hospital, Joint Heidelberg-IDC Translational Diabetes Program, Germany; 9 Institute of Molecular Vascular Medicine, TUM Klinikum, Technical University Munich, Germany; 10 Joint Heidelberg-IDC Translational Diabetes Program, Dept. of Inner Medicine I, Heidelberg University Hospital, Heidelberg, Germany

**Keywords:** Atherosclerosis, CD4⁺ T cells, TBL1X/TBL1XR1, Nuclear receptor coregulators

## Abstract

Atherosclerosis is a long-term complication of obesity and diabetes and as such a key driver of vascular dysfunction and eventually mortality in affected patients. Both aberrant lipid metabolism and inflammatory reactions promote atherosclerotic plaque development in the vessel wall by triggering a cascade of cellular events involving multiple cell types, including smooth muscle cells, monocytic macrophages, and lymphocytes. Despite its eminent impact on human health, molecular drivers of cellular dysfunction in atherosclerosis remain poorly defined and therapeutic options are scarce.

Here we show by single-cell RNA sequencing that the expression of the nuclear receptor co-factors, TBL1X and TBL1XR1, was particularly prominent in the CD4^+^ T cell population of human carotid artery plaques. Indeed, genetic double deletion of TBL1X/TBL1XR1 in CD4^+^ T cells led to a substantial shift from naïve CD44^low^CD62L^hi^ cells to CD44^hi^CD62L^low^ effector and Foxp3^+^ Tregs. CD4^+^ TBL1X/TBL1XR1 KO cells exhibited enhanced cytokine production capacity upon ionomycin/PMA stimulation, correlating with the induction of pro-inflammatory and cytokine-producing transcriptional pathways in these cells. Consistently, transplantation of bone marrow from CD4^+^-specific TBL1X/TBL1XR1 knock out mice into LDLR KO recipients doubled the development of atherosclerotic plaques in the aortic arch compared with wild-type bone marrow transplanted littermates. As TBL1X/TBL1XR1 expression levels were diminished in carotid arteries from patients with advanced unstable plaques compared to stable plaques or healthy controls, these data suggest that aberrant inhibition of TBL1X/TBL1XR1 in CD4^+^ T cells may contribute to the development of atherosclerosis in humans. Restoration of TBL1X/TBL1XR1 functionality may thus serve as a novel, druggable strategy for preventing or limiting atherosclerosis progression.

## Highlights

• Plaque T cells express TBL1X/TBL1XR1; scRNA-seq of human carotid plaques shows enrichment of both cofactors in CD4^+^ T cells.
• CD4-restricted TBL1X/TBL1XR1 loss skews T-cell states; CD4^+^ T-cell depletion expands effector, regulatory cells and cytokine.
• TBL1X/TBL1XR1 control inflammatory and redox programs; transcriptomics show AP-1 and ATF3 pathway activation in CD4^+^ T cells.
• Deficiency promotes atherosclerosis without metabolic change; in LDLR^-/-^ chimeras KO doubles arch lesions, not other sites.
• In unstable human carotid plaques, reduced TBL1X/TBL1XR1 expression highlights these cofactors as potential treatment target.

## 1 Introduction

In Western countries, cardiovascular disease (CVD) remains a critical driver of mortality in an increasingly elderly population. Indeed, obesity and diabetes are the main risk factors and causes for CVD and are expected to further rise within the next decades worldwide [1]. Both obesity and diabetes are characterized by dysfunctional lipid and glucose homeostasis coupled to the manifestation of a low-grade, chronic inflammatory state, eventually leading to irreversible damage within the cardiovascular system. As a reflection of disturbances in systemic energy homeostasis and elevated pro-inflammatory tone, particularly the accumulation of lipid deposits in the arteries and their progressive accretion to intra-luminal plaques, i.e. atherosclerosis imposes a major health issue in subjects with obesity and/or diabetes [2]. This holds particularly true because remission of already established plaques is still not possible to date, thereby defining a major unmet clinical need in cardiovascular and diabetes therapy.

Initiated by inflammatory processes in the endothelial lining of the arteries and a subsequent increase in endothelial permeability as well as expression of endothelial adhesion molecules, low-density lipoprotein (LDL)-bound cholesterol and leukocytes are retained in the sub-endothelial space. There, LDL is modified to oxidized LDL and engulfed by infiltrating monocytes, eventually triggering foam cell formation, and further enhancing inflammation [3]. After initiation, lipids and foam cells continue to accumulate, and additional leukocytes, particularly T-cells, enter the plaque, interacting with the macrophages. T cells are part of the adaptive immune response in atherosclerosis and are activated by various antigen presenting cells (APCs). CD4^+^ T cell subpopulations are the most widely studied subsets in atherosclerosis. They can inhibit the progression of atherosclerosis through immune activation or immunosuppression. Co-stimulatory molecules expressed on the surface of APCs and cytokine mediated crosstalk play a central role in the modulation of the T cell response in atherogenesis [4].

While foam cells frequently undergo apoptosis, thereby leading to the manifestation of a necrotic core, smooth muscle cells also switch from a contractile to a proliferating state and migrate to the plaque region, generating a so-called “fibrous” cap and preventing plaque rupture [5]. Plaque rupture and clotting may then cause clinically relevant vessel closure and myocardial infarction or stroke.

While numerous studies have pinpointed the contribution of individual cell types to atherosclerotic plaque formation, the molecular determinants of intra-cellular, pro-atherosclerotic pathways remain in largely unknown.

Transducin-beta-like, X-linked (TBL1X) and TBL1X-related (TBLXR) 1 represent highly related members of the transcriptional cofactor protein family, originally identified as components of the HDAC3 co–repressor complex [6]. Recent studies have documented the critical role of the TBL1X/TBL1XR1 co-factor complex in tissue-specific and systemic energy homeostasis. High-fat diet-induced inhibition of TBL1X/TBL1XR1 in the liver contributed to hepatic steatosis under obese conditions, driven by the loss of TBL1X/TBL1XR1 co-activator function of the nuclear receptor PPARalpha [7]. Genetic deficiency of TBL1XR1 in adipocytes led to an obese phenotype based on the inhibition of lipid mobilization in these cells [8]. Of note, deficiency of TBL1XR1 not only controlled distinct transcriptional steps in the lipolytic cascade in adipocytes but also triggered the appearance of “crown-like” structures, i.e. the accumulation of immune cells, in the knockout adipose tissue and enhanced pro-inflammatory gene expression [8]. These findings promoted the hypothesis that the TBL1X/TBL1XR1 transcriptional co-factor complex may serve as a point-of-convergence for both metabolic and inflammatory pathways in the control of systemic energy homeostasis and metabolic dysfunction associated with obesity and diabetes.

Here, we show that TBL1X/TBL1XR1 were predominantly expressed in CD4^+^ T cells within human atherosclerotic plaques. T cell-specific genetic knockout of TBL1X/TBL1XR1 in mice led to a marked shift of splenic naïve T cells to activated sub-types with an enhanced pro-inflammatory profile. Bone marrow-transplantation-mediated transfer of TBL1X/TBL1XR1-deficient T cells into atherosclerosis-prone LDLR knockout mice doubled the occurrence of atherosclerotic plaques in the aortic arch of these animals. Indeed, levels of TBL1X/TBL1XR1 were found to be diminished in ruptured vs. stable human plaques, substantiating a critical molecular checkpoint function for TBL1X/TBL1XR1 in T cell-dependent atherogenesis.

## 2 Results

### 2.1 TBL1X/TBL1XR1 are enriched in CD4+ T cells in human carotid artery plaques

**Figure 1:**
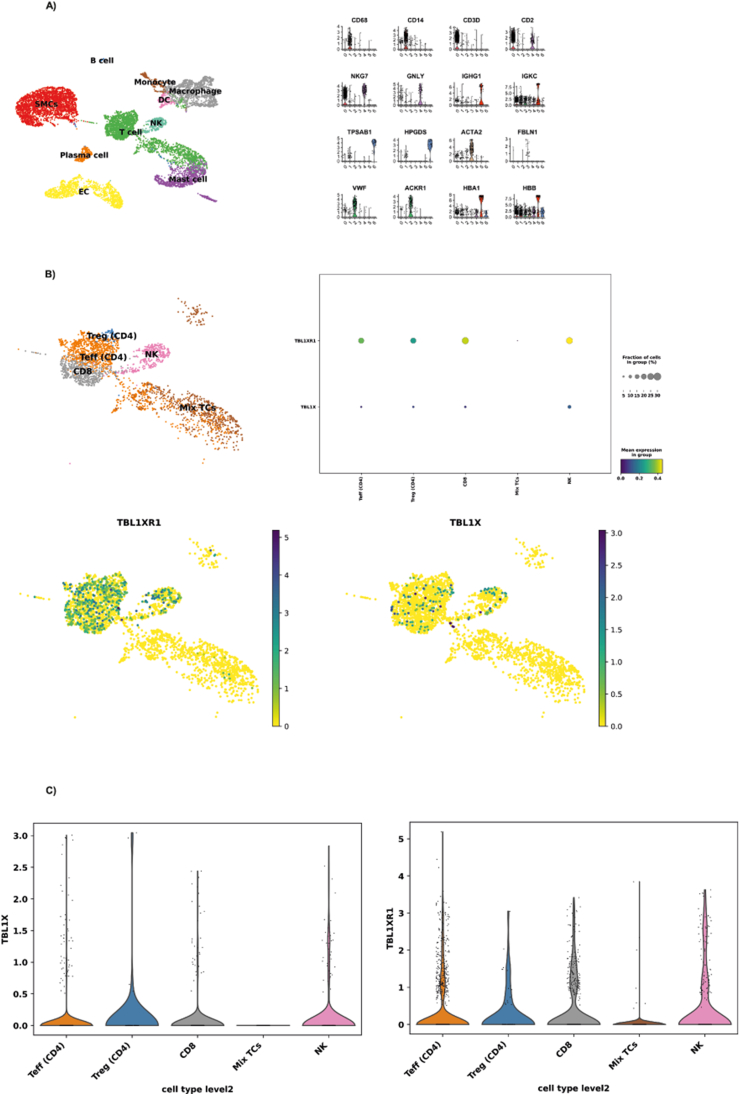
**TBL1X and TBL1XR1 are preferentially expressed in CD4^+^T cells.** Samples obtained from early (control) and advanced (plaque) carotid atherosclerotic lesions collected from 37 patients undergoing carotid endarterectomy. (A) UMAP clustering of carotid artery plaque (CEA patients) of scRNAseq data demonstrated the presence of major plaque-associated cell types. (B) Preferential expression of TBL1X and TBL1XR1 in T cell subtypes of human atherosclerotic plaques. (C) Distribution, expression level and percentage of TBL1X and TBL1XR1 in specific T cell, NK and mast cells.

Given the critical regulatory role of TBL1X/TBL1RX1 under conditions of metabolic and inflammatory stress [7,8], we initially aimed to test the hypothesis that the TBL1X/TBL1XR1 co-factor complex was involved not only in early stages of obesity and pre-diabetes but also in their long-term immuno-metabolic complications. To this end, we interrogated the Munich Vascular Biobank for TBL1X/TBL1XR1 expression levels in human carotid artery plaques. Gene marker-based cell-type annotation of single cell RNA sequencing data demonstrated that all major plaque-relevant cell types could be identified in the plaques, including endothelial and smooth muscle cells, myeloid cells, and leukocytes (Figure 1A). Gene expression analysis revealed that both TBL1X and TBL1XR1 were predominantly expressed in the T cell population within human atherosclerotic plaques with overall higher expression levels of TBL1X as compared with TBL1XR1 (Figure 1B). Notably, TBL1X and TBL1XR1 were particularly enriched in the CD4^+^ T cell sub-population (Figure 1C), arguing for a prominent role of this transcriptional co-factor complex in plaque-associated CD4^+^ T cells.

### 2.2 CD4^+^-specific TBL1X/TBL1RX1 deficiency enhances cytokine production in the splenic T cell population

**Figure 2:**
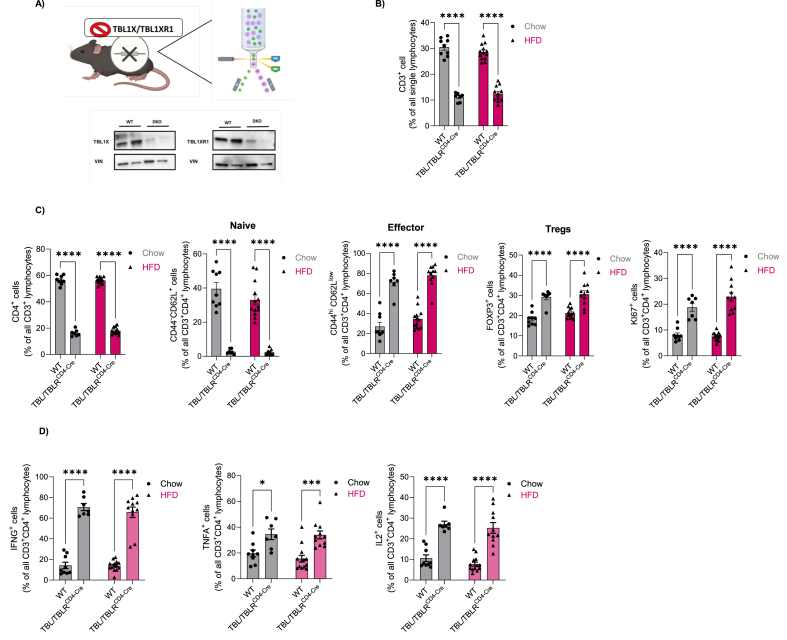
**The depletion of TBL1X/TBL1XR1 in CD4+ T cells increases cytokine production in the splenic T cell population.** (A) Western blot confirmation of double knock out of TBL1X/TBL1XR1 in CD3^+^CD4^+^T cells isolated from spleen and lymph nodes (n = 2). (B) Percentage of CD3+ T cells of all lymphocytes upon TBL1X and TBL1XR1 depletion in chow (WT n = 9, KO n = 7) and HFD-fed (WT n = 13, KO n = 11) mice, assessed by flow cytometry. (C) Percentage of CD4^+^ cells as percentage of all CD3^+^ lymphocytes. Naïve (CD44^low^CD62L^hi^), effector (CD44^hi^CD62L^low^), Tregs (FOXP3^+^) and KI67^+^ cells as percentage of all CD3^+^CD4^+^ T cells, isolated from the spleen and lymph nodes in TBL1X/TBL1XR1 KO settings, in chow and HFD-fed mice (same as in Figure 2B). (D) *Ex vivo* characterization of stimulated CD4^+^ T cells isolated from spleen and lymph nodes for effector cytokine expression (IFNγ, TNFα, IL2), assessed by flow cytometry upon TBL1X/TBL1XR1 depletion (same mice as in Figure 2B). Data are mean ± s.e.m. Two-way ANOVA with Tukey's multiple-comparison *post hoc* test. ∗p < 0.05, ∗∗p < 0.01, ∗∗∗p < 0.001, ∗∗∗∗p < 0.0001.

To test a potential functional role of T cell TBL1X/TBL1XR1 in metabolic disease and atherosclerotic plaque formation, we generated T-cell-specific TBL1X/TBL1XR1 knockout animals by crossing animals carrying the CD4^+^-specific Cre driver (B6.Cg-Tg (Cd4-cre)1Cwi/BfluJ) with TBL1X/TBL1XR1 double-floxed mice, producing TBL1/XR1 knockout in both CD4^+^ and CD8^+^ T cells. As TBL1X and TBL1XR1 play highly redundant roles in tissue-specific transcriptional control [7], we aimed to avoid potential functional compensation by employing a double gene knockout strategy. Western Blot analysis of TBL1X and TBL1XR1 protein expression in isolated CD4^+^ T cells verified an almost complete depletion of TBL1X/TBL1XR1 expression in these cells upon Cre-mediated recombination (Figure 2A).

To explore a potential metabolic role of TBL1X/TBL1XR1 -depleted T cells, both male and female wild-type, and KO littermates were placed on either chow or high fat diet (HFD) (60% calories from fat) for 14 weeks (wks), starting at 6 wks of age. HFD feeding triggered the expected increase in body weight and body fat content and led to impairments in glucose and insulin tolerance as compared to chow-fed animals. No obvious differences in metabolic phenotype were observed between KO and wild-type animals in both sexes (Sup. Figure 1), suggesting that the TBL1X/TBL1XR1 co-factor complex in CD4^+^ T cells does not play a dominant role in normal physiological control of energy homeostasis under conditions of early pre-diabetic obesity. Indeed, plasma levels of glucose, triglycerides, and cholesterol as well as body weight remained unchanged between the genotypes, irrespective of the type of dietary intervention (Sup. Figure 1).

To further test whether TBL1X/TBL1XR1 double deficiency elicited an impact on actual T cell function, we isolated lymphocytes from the spleens of experimental animals both under chow and HFD conditions at the age of 20 wks. Double KO of TBL1X/TBL1XR1 led to a threefold reduction in CD3^+^ lymphocytes (as percentage of all lymphocytes) with no significant differences between male and female animals under both chow and HFD conditions (Figure 2B, Sup. Fig.5A,B). Further, we observed a significant reduction in the CD4^+^, but not CD8^+^ T cell population (% CD4^+^ of CD3^+^ cells) (Fig.2C, Sup. Fig.2A). Naïve CD4^+^ as well as naïve CD8^+^ cells were nearly absent in KO mice as compared to wild-type littermates (Fig.2C, Sup. Fig 2B). In contrast, CD4^+^ effector cells (CD44^hi^ CD62L^low^)) and FoxP3^+^ regulatory T cell subpopulations were substantially elevated, correlating with the overall induction of proliferative Ki67^+^ T cells in KO male mice (Figure 2C). Furthermore, we observed a significant increase in the CD4^+^ central memory (CD44^low^CD62L^hi^) T cell population upon TBL1X/TBL1XR1 KO, in chow fed diet group (Sup. Figure 2C).

Remarkably, both CD4^+^ and CD8^+^ TBL1X/TBL1XR1-deficient T cells exhibited strikingly increased expression of the pro-inflammatory cytokines IFN-γ and IL-2 following ionomycin/PMA stimulation, whereas TNF-α was significantly upregulated in CD4^+^ T cells and showed a non-significant but consistent trend toward increased expression in CD8^+^ T cells (Figure 2D, Suppl. Fig. 2D). These findings suggest that loss of TBL1/TBLR1 co-activator function in T cells promotes a shift from a naïve toward an effector phenotype with an enhanced pro-inflammatory profile.

### 2.3 TBL1X/TBL1XR1 control cytokine and inflammatory molecular pathways in T cells

**Figure 3:**
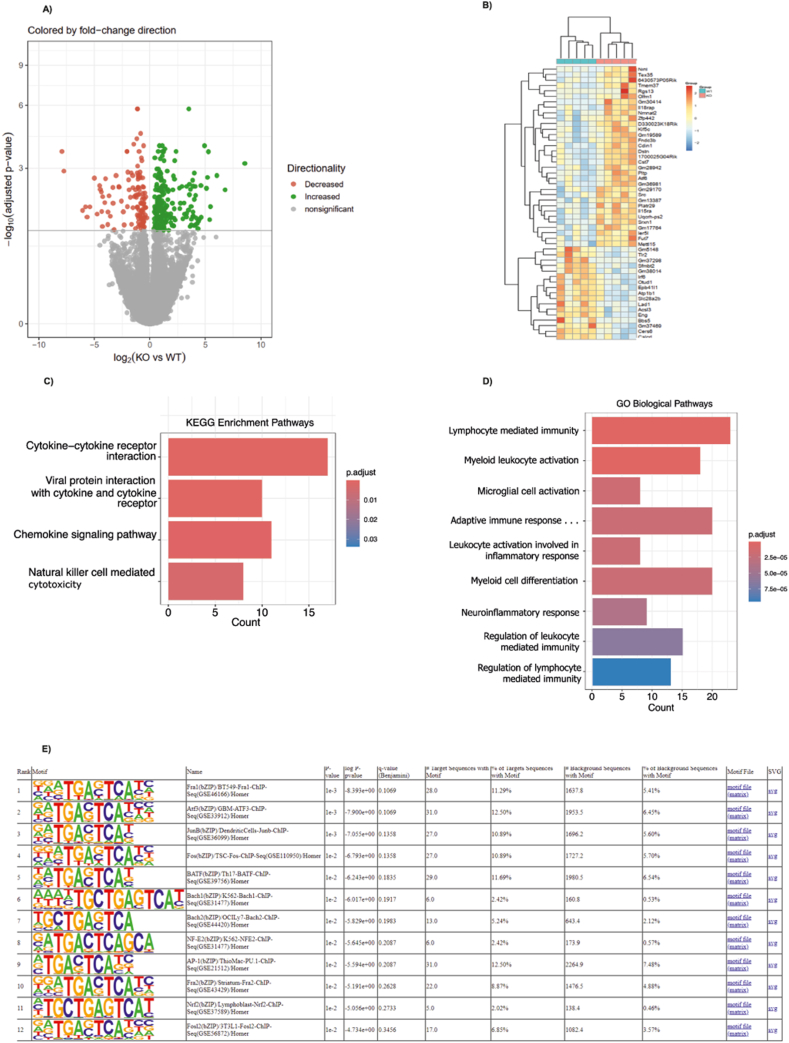
**TBL1X/TBL1XR1 deficiency triggers distinct transcriptomic responses in CD4+ T cells.** (A) Clear transcriptomic separation between TBL1X/TBL1XR1 KO (n = 6) and WT(n = 5) cells. (B) Genetic deficiency of TBL1X/TBL1XR1 leads to a particular regulation of distinct gene sets. (C) Differentially expressed genes (DEGs) in TBL1X/TBL1XR1-deficient cells. (D) Gene ontology (GO) term analysis in TBL1X/TBL1XR1-deficient cells. (E) Transcription factor motif analysis in a gene signature regulated by the depletion of TBL1X/TBL1XR1 in CD4+ T cells.

To identify the mechanisms by which the TBL1X/TBL1XR1 co-factor complex mediates its impact on T cell phenotypes and pro-inflammatory profiles, we performed RNA sequencing from wild-type and KO CD4^+^ T cells isolated from the spleen. RNA sequencing analysis showed that 326 genes were differentially expressed between phenotypes, correlating with a clear transcriptomic separation of wild-type and KO cells in a principal component analysis (Figure 3A; Sup. Figure 3A). In line with the pro-inflammatory phenotype (Figure 2), genetic deficiency of TBL1X/TBL1XR1 led to a particular regulation of genes involved in inflammatory and cytokine-related pathways (Figure 3B,C). Indeed, within the GO term analysis, leukocyte activation/immunity pathways were amongst the most significantly regulated biological processes regulated by the absence of TBL1X/TBL1XR1 (Figure 3D), demonstrating that the TBL1X/TBL1XR1-dependent conversion of naïve to effector T cell populations with enhanced pro-inflammatory capacities emerges from the transcriptional control of related genetic networks, responsible for T cell phenotype shaping. Of note, subsequent transcription factor motif analysis using RNA seq data from TBL1X/TBL1XR1 wild-type and KO CD4^+^ T cells showed that the TBL1X/TBL1XR1 complex predominantly altered the activity of AP-1 and ATF3 transcription units, previously known to control essential components of inflammation and diabetes-driven atherogenesis (Figure 3E), [[9], [10], [11]].

Consistent with these findings, network integration of the differentially expressed genes revealed that cytokine- and lipid-responsive regulators formed highly connected clusters converging on TBL1X/TBL1XR1 (Sup.Fig.3B). This suggested that plaque-associated inflammatory and metabolic signals act as upstream drivers of TBL1X/TBL1XR1-dependent transcriptional programs in T cells. Moreover, we also detected a redox-related transcriptional shift in KO CD4^+^ T cells, characterized by increased expression of the NRF2-responsive antioxidant gene *Srxn1* and reduced expression of *Bach2* and *Pak1* (Sup. Fig.3C). This pattern suggests disrupted oxidative-stress handling in the absence of TBL1X/TBL1XR1, complementing the pro-inflammatory transcriptomic changes described above.

### 2.4 TBL1X/TBL1RX1 CD4^+^-specific KO induces atherosclerotic plaque development in bone marrow-transplanted LDLR KO animals

**Figure 4:**
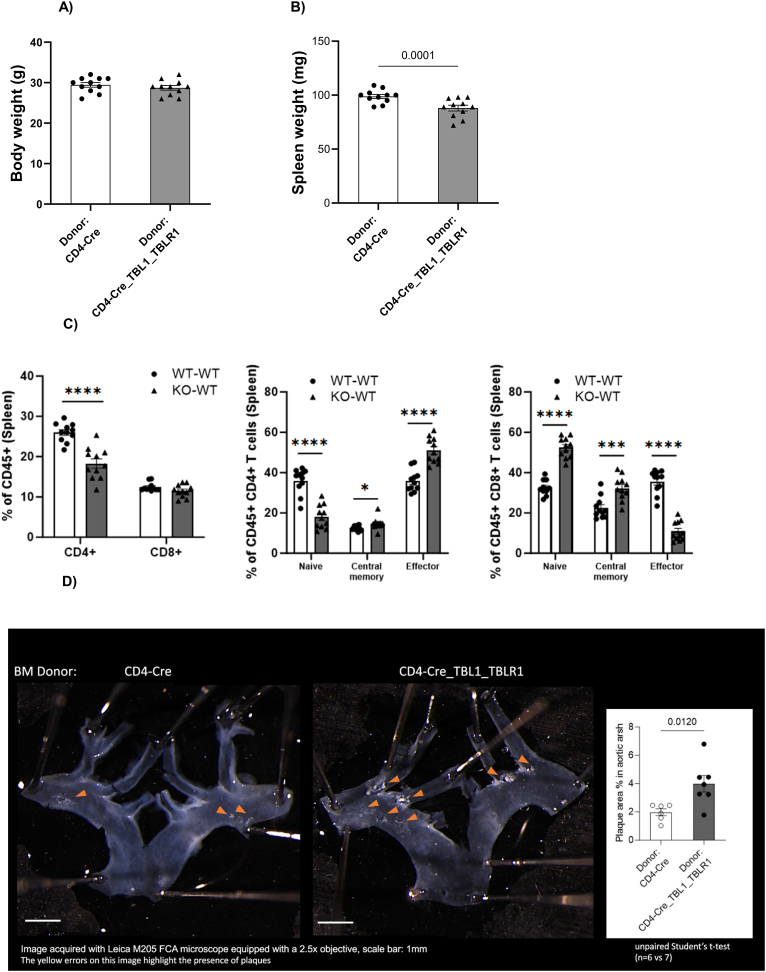
**Deletion of TBL1X/TBL1XR1 in T cells enhances atherosclerosis.** (A) Body weights of WT and KO cells transplanted animals after a 10-week western diet (WD). (B) Spleen weight in transplanted mice after 10 weeks WD. (C) Flow cytometric analysis of splenic CD4+ and CD8+ T cells in transplanted mice after 10 weeks WD. (D) Atherosclerotic plaques in the aortic arch of transplanted mice assessed by en face analysis of aortic lesions after 10 weeks WD. All data shown in A-D were measured at the study endpoint, following WT or KO bone marrow transplantation, 6 weeks recovery and 10 weeks WD.

Our data thus far demonstrated a significant regulatory role of the TBL1X/TBL1XR1 co-factor complex in the control of T cell development and CD4^+^ T cell pro-inflammatory gene expression. This supported the notion that TBL1X/TBL1XR1 in T cells may have a relevant impact on immuno-metabolic disease conditions, typically important in the more long-term stages of obesity and diabetes-related complications but rather less significant in the earlier, pre-diabetic conditions imposed by simple HFD feeding. Of note, decreased numbers of CD4^+^ naïve T cells have previously been implicated in increased abundance of cardiovascular events in patients [12]. To test this assumption experimentally, we employed bone marrow transplantation from TBL1X/TBL1XR1 CD4^+^ wild-type and KO animals into irradiated LDLR KO mice, a standard model for diet-induced atherosclerosis development, triggered by metabolic but also -partially- T cell-dependent inflammatory reactions [13]. Following six weeks of rest after irradiation, transplanted animals were placed on a western diet for an additional 10 weeks. At the end of the experimental period, wild-type and KO transplanted mice did not differ in body weight or other metabolic parameters (Figure 4A) but displayed a significant reduction in spleen mass (Figure 4B). Characterization of lymphocytes isolated from spleens of these LDLR KO animals recapitulated the CD4^+^ T cell phenotype from the initial HFD experiment (Figure 2), including the reduction in overall CD4^+^ and particularly naïve CD4^+^ T cells with the concomitant induction of CD4^+^ effector cells (Figure 4C), thereby validating our original findings in an independent experimental setting. Of note, under these experimental conditions, CD8^+^ T cells displayed a somewhat opposite phenotype, most likely explained by a CD45+ gating strategy in contrast to CD3+ gating in the previous experiment (Figure 4C).

**Figure 5:**
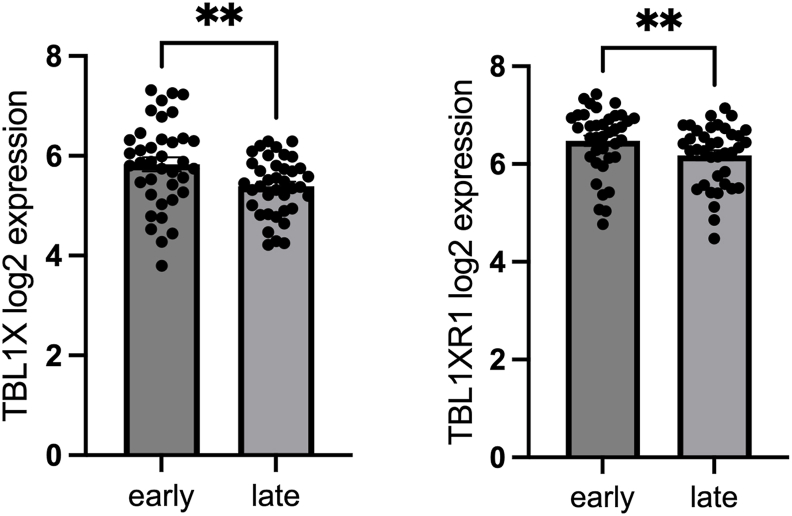
**TBL1X/TBL1XR1 expression levels are diminished in human atherosclerotic plaques and correlate with disease severity.** RNA sequencing of whole tissue samples derived from paired early and advanced (n = 37) carotid artery plaque samples shows a significant decrease in both TBL1X and TBL1XR1 expressions. Data presented as mean +SEM. ∗∗p < 0.01. Significance was determined using two-tailed paired Student's T-test.

Remarkably, en face aortic lesion quantification demonstrated a doubling of atherosclerotic plaques in the aortic arch of KO bone marrow transplanted mice as compared with wild-type transplanted littermates (Figure 4D). In contrast, no major changes in plaque development were identified in the descending aortic regions (Sup. Figure 4). These data demonstrated that the transcriptional activity of the TBL1X/TBL1XR1 co-factor complex in T cells exerts a critical, anti-inflammatory role in the prevention towards diet- and obesity-induced atherosclerosis.

### 2.5 TBL1X/TBL1XR1 expression is diminished in human carotid plaques

The animal data implied that the maintenance of T cell-TBL1X/TBL1RX1 was required for the prevention of inflammatory atherosclerosis, functionally validating a negative impact on atherosclerotic phenotypes upon loss and/or inhibition of TBL1X/TBL1XR1 in plaque-associated T cells. Indeed, bulk RNA sequencing of healthy control carotid arteries and advanced stable plaques from patients with CVD demonstrated a significant reduction of both TBL1X and TBL1XR1 gene expression in the advanced plaque conditions (Figure 5). This supports the notion that low-level TBL1X/TBL1XR1 expression is associated with unfavorable atherosclerotic phenotypes also in humans.

## 3 Discussion

This study tested the hypothesis that the TBL1X/TBL1XR1 transcriptional co-factor complex serves as a point of convergence for both metabolic and inflammatory pathways in systemic energy homeostasis and metabolic dysfunction. The metabolic function of these transcriptional co-factors has been well established in a series of previous studies. Inhibition of TBL1X/TBL1XR1 in response to the development of fatty liver disease contributed to lipid accumulation, related to the inhibition of mitochondrial respiration [7]. In line with a metabolic function, TBLX1 also determined the activity of the PI3K/Akt pathway in pancreatic exocrine tumor cells [14]. In white adipose tissue, TBL1XR1 but not TBL1X was found to be a key determinant of lipolytic pathway activity, driving the manifestation of an obese phenotype upon WAT-specific gene ablation [8]. Of note, under these conditions, TBL1XR1 deficiency triggered the appearance of “crown-like” structures, i.e. the accumulation of immune cells, in the knockout adipose tissue and enhanced pro-inflammatory gene expression in WAT [8], indicating a potential immune-metabolic integrative function of these cofactors.

The current study now provides evidence for this notion, demonstrating a key anti-inflammatory role of the TBL1X/TBL1XR1 complex in T cell-dependent atherogenesis. Loss of TBL1X/TBL1XR1 complex activity led to a shift from naïve to effector T cell subtypes, characterized by a pro-inflammatory gene expression profile. As atherogenesis is generally driven by combinatorial metabolic and inflammatory insults to the vessel wall, the molecular control of T cell function by TBL1X/TBL1XR1 elicits a critical checkpoint role for vessel health under physiological conditions, and is underlined by the heightened severity of plaque development upon TBL1X/TBL1XR1 loss-of-function.

The observed rise in Foxp3^+^ Tregs despite heightened plaque inflammation is consistent with niche-specific and context-dependent Treg adaptation described in non-lymphoid tissues [15,16]. During hypercaloric challenges, Tregs decline in metabolic organs such as visceral adipose tissue, whereas Tregs in lymphoid organs often expand or are retained, likely reflecting inflammation-induced proliferation and altered trafficking [[17], [18], [19]]. In our model, TBL1X/TBL1XR1 loss in T cells coincided with increased plaque inflammation and burden, reduced splenic cellularity, and a relative decrease of conventional CD4^+^/CD8^+^ cells in lymphoid tissue, suggesting preferential recruitment of effector T cells to plaques while Tregs accumulate or are retained in lymphoid compartments. We therefore interpret the lymphoid Treg increase as a systemic regulatory response rather than evidence of enhanced intraplaque immune suppression.

The observation of preferential expression of TBL1X/TBL1XR1 in CD4+ T cells in human carotid artery plaques suggests that these transcription co-factors might play a significant role in human atherosclerotic plaques by shaping the T cell mediated immune response and by inducing changes in T cell profile. Since they are involved in the NCoR/SMRT complex together with HDAC3 [20], TBL1X and TBL1XR1 contribute to the stoichiometric status of the complex. Therefore, depletion of TBL1X and TBL1XR1 might disrupt this stability, resulting in deregulation of the genes required for T cell-mediated immune response along with T cell profile itself.

The TBL1X/TBL1XR1 complex elicits pleiotropic roles in terms of its transcriptional co-activating functions [21,22]. In line with the transcription factor motif analyses in T cells, transcription factor motifs such as Fra1, Fos, JunB, Batf, belonging to the AP-1 family [23] and ATF3 are highly represented in TBL1X/TBL1XR1 target gene sets suggesting that depletion of TBL1X and TBL1XR1 increases AP-1-driven gene expression, resulting in pro-inflammatory responses in T cells. With network analysis, we revealed that plaque-derived inflammatory and lipid signals form tightly connected regulatory clusters converging on TBL1X/TBL1XR1 (Sup. Fig.3B). This supports the hypothesis that cytokines such as IL-6, IL-1β, TNF, IFN-γ and IL-17A together with lipid-sensing nuclear receptors may generate upstream signals that enhance TBL1X/TBL1XR1 activity in plaque-infiltrating T cells.

Moreover, overrepresentation of Nrf2, Bach1 and Bach2, which have roles in inflammatory pathways upon oxidative stress [24], imples that TBL1X and TBL1XR1 depletion potentially induces oxidative stress affecting T cell redox balance, eventually contributing to the observed inflammatory phenotypes. We also identified an oxidative-stress signature in KO T cells, with Srxn1 upregulated and Bach2 and Pak1 downregulated (Sup.Fig.3C), arguing for a disrupted redox balance and increased oxidative pressure in the absence of TBL1X/TBL1XR1, known to amplify T-cell inflammatory activation in atherosclerosis. In the future, it will be important to determine whether these predicted upstream signaling pathways determine TBL1X/TBL1XR1 gene expression levels in T cells and may contribute to the observed drop in expression levels in unstable vs. stable plaques in humans.

Beyond their co-factor function for PPAR nuclear receptor complexes in liver and adipose tissue, the TBL1X/TBL1XR1 complex thus appears to interact with a distinct, and T cell-specific set of DNA-binding partners in these cells. Overall, this underlines the pleiotropic molecular function of this complex and its dependence on the specific cellular environment.

Interestingly, while both TBL1X and TBL1XR1 have been initially described as functionally redundant [25], they may have independent functions in certain settings [26]. Indeed, while TBL1XR1 KO in WAT was sufficient to impair lipolysis [8], it remains to be clarified whether only double activation of TBL1X and TBL1XR1 or whether even single activation will be required to maintain anti-inflammatory properties of T cells, which will eventually become relevant when evaluating a therapeutic potential of this complex. In any case, the question of tissue-specific redundancy remains unclear, and its molecular basis will have to be explored in more detail in the future.

Overall, atherosclerosis and associated vascular complications remain a major determinant of mortality in obese and diabetic subjects. As it is still not possible to drive existing plaques into remission, the prevention of atherogenesis is at the forefront of clinical efforts in this field. Our study now establishes a novel molecular rationale for T cell-specific pro-inflammatory functions in this disease and suggests that the maintenance of proper TBL1X/TBL1XR complex activity may contribute to preventive anti-atherogenic measures in the future. As TBL1X/TBL1XR1 inhibition correlates with advanced plaque phenotypes in humans, pre-clinical and clinical data align well to stimulate corresponding efforts in future drug development studies.

## 4 Material and methods

### 4.1 Human samples

#### 4.1.1 RNA sequencing (RNAseq)

RNA libraries were prepared and sequenced on the Illumina NovaSeq 6000 platform (Illumina, San Diego, USA) using RNA extracted from non-diseased (control) and diseased (plaque) tissue biopsies obtained from 37 patients undergoing carotid endarterectomy. Total RNA was processed using poly-T oligo-attached magnetic beads to isolate messenger RNA. Fragmentation was followed by first-strand cDNA synthesis using the TruSeq Stranded Total RNA Kit and the TruSeq Stranded mRNA Kit (Illumina). The second-strand cDNA synthesis was performed with dUTP incorporation. Subsequent steps included end repair, A-tailing, adaptor ligation, size selection, amplification, and purification to generate the final directional library. Library quantification was assessed using Qubit and real-time PCR, while size distribution was analyzed with a Bioanalyzer. Raw sequencing data (FASTQ files) were processed by Fios Genomics (Edinburgh, UK). Quality control (QC) was performed on the raw reads, and alignment statistics were obtained using the STAR aligner. Sequencing length distributions were consistent and of high quality across all samples, allowing their inclusion in downstream analyses.

### 4.2 Single-cell RNA library preparation, sequencing, and data analysis

Single-cell RNA sequencing (scRNA-seq) libraries were prepared using the 10x Genomics Chromium platform. Cells were encapsulated within a 10x Genomics microfluidics Chip G, where they were combined with barcoded oligo-dT-containing gel beads via the Chromium Controller. Following clean-up of Gel Beads in Emulsion (GEMs), cDNA amplification was performed, and a 3′ gene expression library was generated according to the manufacturer's protocol (CG000204 Rev D). For scRNA-seq data analysis, the Seurat package (version 4.1.1) was utilized within RStudio (version 1.4.1717). The dataset can be accessed publicly under the accession code GSE247238. The final analysis included samples from nine patients, each contributing both early-stage and advanced-stage samples. Genes detected in fewer than five cells were excluded from downstream analysis. Additionally, stringent quality control measures were applied, filtering out cells with mitochondrial read content exceeding 15%, a total UMI count greater than 20,000, fewer than 100 detected genes, or more than 3,000 genes. Cell cycle phase scores, including S and G2M phases, were assigned using Seurat's CellCycleScoring function. To minimize variability, the SCTransform normalization method was applied. Dimensionality reduction was performed using Uniform Manifold Approximation and Projection (UMAP) with default settings, enabling visualization of the primary characteristics of each cell cluster.

#### 4.2.1 Animal studies

Experiments involving mice were conducted using C57BL/6N mice. The animals were maintained under standard conditions, including a 12-hour light–dark cycle at a temperature of 22 °C, with unrestricted access to standard rodent chow and water unless otherwise specified. All animal care and experimental procedures were carried out in compliance with the guidelines of the institutional animal welfare officer and received approval from local regulatory authorities (Breeding Licenses 16–117 and 21–133) for T cell knock out animals, ROB-55.2.2532.Vet_02-18-114 for bone marrow transplantation).

T-cell-specific TBL1X/TBL1XR1 knockout animals were generated by crossing animals carrying the CD4^+^-specific Cre driver (B6.Cg-Tg(Cd4-cre)1Cwi/BfluJ) with TBL1X/TBL1XR1 double-floxed mice, producing TBL1X/TBL1XR1 knockout in both CD4^+^ and CD8^+^ T cells. To study the phenotypic effects of genetically modified mice, 6 weeks old male and female mice were put in either 60% HFD or chow diet for 14 weeks.

Both male and female mice were euthanized by cervical dislocation for T cell knock out studies. For bone marrow transplantation study, male mice were used, and they were euthanized under deep anesthesia with ketamine/xylazine, in order to collect blood via cardiac puncture and perfusion of organs before harvesting the heart, aorta, spleen, femurs, and livers.

#### 4.2.2 Intraperitoneal glucose tolerance test (ipGTT)

Mice were fasted for 6 h, and basal blood glucose was recorded using an automatic glucose monitor. Then they were i.p.-injected with 2 g/kg body weight glucose and blood glucose levels were determined 15,30, 60 and 120 min after injection.

#### 4.2.3 Intraperitoneal insulin tolerance test (ipITT)

Mice were fasted for 6 h before the experiment. Body weight and baseline blood glucose levels were measured before the experiment. Mice were then administered an intraperitoneal injection of insulin at a dose of 0.7 U/kg, diluted in 0.9% NaCl. Blood glucose levels were monitored at 15, 30, 45, 60, 90, and 120 min following the injection.

#### 4.2.4 Serum analysis

Serum was prepared by allowing the collected blood to sit at room temperature for 5 min, followed by centrifugation at 10,000 g for 10 min at 4 °C. The resulting serum was transferred into a clean tube, rapidly frozen using liquid nitrogen, and stored at −80 °C until analysis. Levels of glucose, cholesterol and triglycerides were measured using the Beckman Coulter AU480 Chemistry Analyzer.

#### 4.2.5 Isolation of splenic and lymph node cells

To obtain single cell suspensions, spleens and lymph nodes were mashed through a 70 μm or 100 μm Falcon cell strainer. RPMI medium (with 10% FCS, without 2-mercaptoethanol) was continuously added using a 1 mL pipette to facilitate cell collection. Cells were pelleted at 400 g for 5 min at 4 °C. For splenic cells, 1 mL of ACK buffer was added to lyse red blood cells (RBCs). After a 2-minute incubation at room temperature, the lysis was stopped by adding 9 mL of RPMI without FCS or 2-mercaptoethanol. Lymph node cells were resuspended in 2 mL of complete RPMI. Both samples were centrifuged again at 400g for 5 min at 4 °C. For flow cytometric analysis of splenic T cells, cells were filtered through a 40 μm cell strainer and suspended in FACS buffer. For magnetic isolation of CD4+ and CD8+ T Cells, cells were resuspended in 10 mL of complete RPMI and counted. Each sample was divided into two parts for subsequent CD4^+^ and CD8^+^ T cell isolation.

#### 4.2.6 Flow cytometry

The following antibodies were used for flow cytometry (reactivity, fluorochrome, clone, manufacturer): CD11b, Pacific Blue, M1/70, BioLegend; CD11c Brilliant Violet 421, N418, BioLegend; B220 Pacific Blue, RA3-6B2, BioLegend; CD14, V450, rmC5-3, BD Biosciences; F4/80, Pacific Blue, BM8, BioLegend; CD3, PerCP-Cy5.5, HIT3a, BioLegend; CD8a, Brilliant Violet 605, 53–6.7, BioLegend; CD4, Alexa Fluor 700, RM4-5, BioLegend; CD3, PE-CF594, 145-2C11, BioLegend; CD62L, BV510, Biozol Diagnostica; CD44, PE,anti-mouse/human CD44 clone [IM7], Biozol Diagnostica 103024; Ki67, PerCP-Cy5.5,clone 16A8,Biolegend; TNFA, PE-Cy7, MAb11, BioLegend; IFNG, APC, 4S.B3, BioLegend; IL2, PE/Dazzle594, MQ1-17H12, BioLegend; FOXP3, FITC, FJK-16s, eBioscience; CD45, BV605,30-F11, BioLegend; CD8,PECy7,53–6.7, eBioscience; CD4, APCy7,GK1.5, BD; CD3, BUV737,17A2, BD; CD44, APC, IM7, Biolegend and CD62L, FITC, MEL14,BD.

All single cell suspensions were incubated with Fc blocking reagent (BD Pharmingen) or anti-mouse CD16/CD32 (clone 93, eBioscience) for 10 min to prevent the nonspecific binding of antibodies, and thereafter incubated with fluorochrome-labelled antibodies for surface staining on ice or at 4 °C in the dark for 30 min. To detect intracellular protein expression, T cells were fixed and permeabilized using the Foxp3 Staining Buffer Set (eBioscience, San Diego, CA, USA) after surface staining as recommended by the manufacturer. To determine intracellular cytokine expression, cells were stimulated for 4h with phorbol 12-myristate 13-acetate (PMA, 50 ng/mL, Abcam, Cambridge) and ionomycin (1 μg/mL, Biomol GmbH, Hamburg) at 37 °C, and GolgiPlug (BD) was added for the last 2 h at 37 °C before flow cytometric staining and cell fixation using the Cytofix/Cytoperm Plus Kit (BD). Cells were passed through a 40 μm cell strainer (NeoLab, Heidelberg, Germany) to remove large debris. Cells were acquired on a BD FACSAriaIII flow cytometer using FACSDiva software V6.1.3 (Beckton Dickinson) or LSR FORTESSA (BD) was used for flow cytometry. Doublets were excluded based on SSC-A vs. SSC-W and FSC-A vs. FSC-W or FSC-A vs. FSC-H plots. Dead cells were excluded using the fixable viability dye eFluor450 (Invitrogen). Samples were analyzed using FlowJo software v10.8.0 (TreeStar Inc., Ashland, OR, USA).

After blocking non-specific binding with Fc block, spleen cells were stained with the following antibodies: and incubated for 30 min 4 °C, then washed and suspended in FACS buffer for measuring.

### 4.3 Magnetic isolation of CD4^+^ and CD8^+^ T cells

Cell pellets were resuspended in 40 μL RPMI per 10^7^ total cells, followed by the addition of 10 μL Biotin-Antibody Cocktail per 10^7^ total cells. The mixture was incubated for 5 min at 4 °C, then filled up to 10 mL with RPMI (without FCS and 2-mercaptoethanol) and centrifuged at 400g for 5 min at 4 °C. The pellet was resuspended in 30 μL RPMI per 10^7^ total cells, and 20 μL of Anti-Biotin MicroBeads were added per 10^7^ total cells. After mixing thoroughly, the suspension was incubated for 10 min at 4 °C. The cell suspension was then diluted to 10 mL with RPMI and centrifuged again at 400g for 5 min at 4 °C. The supernatant was discarded, and the cell pellet was resuspended in 5 mL RPMI. Magnetic separation columns were prepared by rinsing with 3 mL complete RPMI immediately before adding the cell suspension. The cell suspension was loaded onto the column, followed by rinsing the Falcon tube with 2 mL RPMI to collect all cells. Flow-through was collected, and cells were counted. Collected cells were centrifuged at 400g for 5 min at 4 °C, and the supernatant was removed carefully. For downstream functional assays, a 24-well plate coated with appropriate reagents was prepared by washing three times with PBS. The cell pellet was resuspended in 500 μL of RPMI complete and counted again. A total of 0.5 × 10^6^ cells were seeded per well in medium supplemented with 30 U/mL IL-2.

#### 4.3.1 Western Blot analysis

Isolated CD3^+^CD4^+^T cells were harvested in RIPA buffer (Sigma, R0278) having 1x protease inhibitor cocktail (PIC) (Sigma, Munich), 1x phosphatase inhibitor cocktail (Sigma, Munich). 10 μg protein was loaded to 10 % SDS-polyacrylamide-gels and blotted to nitrocellulose membranes. Western blotting was performed using primary antibodies against, TBL1X (Proteintech, 13540-1-AP), TBL1XR1 (Santa Cruz, sc-517365), VIN (abcam,129002).

#### 4.3.2 RNAseq analysis of mouse samples

For bulk RNA sequencing, splenic single cell suspensions were obtained according to the above-mentioned protocol. 200 μL of cell suspension were stained with the following antibodies CD45, CD19, CD3 and zombie green as a live/dad stain. 10,000 Live CD45^+^CD19-CD3^+^ were sorted directly into 100 μL of Buffer RLT Plus (Qiagen, 1053393) using BD FACSAria III. RNA isolation was done using the Qiagen RNA isolation kit Micro, RNA integrity number (RIN) was measured using the Agilent 2100 Bioanalyzer. RNAs with a RIN value > 7 were selected for mRNA sequencing (poly-A selected). The libraries were prepared using the non-stranded SMART-Seq v4 Plus kit (Takara), following the kit's instructions. After a final QC, the libraries were sequenced in a paired-end mode (2x100 bases) in the Nextseq1000 sequencer (Illumina) with a depth of ≥30 Million reads per sample. The adapter sequence from the raw files was removed by using Cutadapt 4.1. Raw counts were then aligned to the mouse reference genome (mm39) using STAR 2.7.10a. Any genes that have no transcript detected in any samples were removed. Data normalization and differential expression analysis were performed using the DESeq2 (v3.14) R-Bioconductor. A statistical threshold was set to the adjusted p-value (Padj) < 0.05 and the effect size was set to a log2 fold change (FC) of < −0.5 or >0.5. For the KEGG pathway and GO analysis, the enrichKEGG and enrichGO function of R clusterProfiler package were used respectively.

### 4.4 Bone marrow transplantation

To test for the role of TBL1X/TBL1XR1 gene in atherosclerosis progression, bone marrow cells were transplanted from TBL1X/TBL1XR1 CD4+ wild-type or KO male mice into lethally irradiated LDLR^*−/−*^ mice. Donor and recipient mice were 7–8 weeks old on the day of transplantation. LDLR^*−/−*^ recipients received two irradiation doses of 5G (Faxitron CP160) with the second dose given 16 h after the first irradiation. Femurs and tibias were collected from donors and further extraction of bone marrow cells was done in sterile conditions (cell culture biosafety BSL2 cabinet), Cells were flushed from bones of at least two mice per group, pooled and counted using trypan blue and TC20 automated cell counter (BioRad). Then 2.5 × 10^6^ cells were transplanted intravenously through tail injection into each recipient. Recipients were provided with mashed food for 10 days and antibiotics for one month. After 6 weeks of recovery, the diet was changed to a western diet (WD) for 10 weeks. Recipient mice were randomly assigned to experimental groups before transplantation. During bone marrow injection, follow-up and euthanasia, cages from different groups were mixed and processed in alternating order to avoid systematic handling bias.

### 4.5 Aorta and arch en face preparation and lesion quantification

Aortic arch and abdominal aortas were excised following euthanasia and heart perfusion with 10 mL of pre-cooled PBS. The isolated vessels were placed in 1.5 mL Eppendorf tubes with 1% PFA solution overnight. After fixation, the vessels were transferred to a petri dish containing PBS. Sequentially, the vessels were carefully opened under a dissecting microscope (Leica), with the removal of fat and connective tissue according to Pei-Yu Chen et al. [27] Subsequently, the opened aorta and arch was fixed with minutiae pins on a rubber plate.

For imaging of the vessels, a Leica M205 FCA microscope equipped with a 2.5X objective was used. For lesion quantification, the ImageJ software was employed to delineate lesion areas using the “polygon selection” tool. Subsequently, the selected areas underwent analysis within the Region of Interest (ROI) management function through measurement and recording. The relative plaque percentage was then determined by normalizing the plaque area to the vessel area. At tissue collection, each sample was given a numerical code. Embedding, sectioning, staining, imaging and quantification of plaque area and collagen content were performed on coded sections with the investigator blinded to genotype and treatment, and codes were only broken after all analyses were completed.

### 4.6 Statistical analysis

Results from biological replicates were expressed as mean ± standard error of the mean (s.e.m.). All statistical analyses were performed using GraphPad Prism 10.2.0. Normal distribution was assumed in all experiments. Significance was tested using unpaired Student's t-test, multiple unpaired t-tests, one-way analysis of variance (ANOVA), paired two-way ANOVA, linear regression analysis and mixed effects analysis as indicated in the figure legends. When comparing two variables, Tukey's or Šidák's post hoc tests were applied for correction. P < 0.05 was considered statistically significant.

## CRediT authorship contribution statement

**Sahika Cingir Koker:** Conceptualization, Data curation, Formal analysis, Investigation, Methodology, Project administration, Validation, Writing – original draft, Writing – review & editing, Supervision. **Amit Mhamane:** Data curation, Methodology, Visualization. **Julia Geppert:** Data curation, Formal analysis, Validation, Investigation, Methodology. **George Shakir:** Formal analysis, Investigation, Methodology. **Raquel Guillamat-Prats:** Formal analysis, Investigation, Methodology. **Bingni Chen:** Methodology, Formal analysis, Investigation. **Pernilla Katra:** Methodology, Formal analysis, Investigation. **Martina Geiger:** Formal analysis, Investigation, Methodology. **Foivos-Filippos Tsokanos:** Investigation, Methodology, Supervision, Data curation, Formal analysis. **Gretchen Wolff:** Formal analysis, Investigation, Methodology, Writing – review & editing. **Julia Szendrödi:** Conceptualization, Resources, Writing – original draft, Writing – review & editing. **Maria Rohm:** Conceptualization, Resources, Writing – original draft, Writing – review & editing, Funding acquisition. **Carolin Daniel:** Conceptualization, Resources, Writing – original draft, Writing – review & editing. **Lars Maegdefessel:** Resources. **Sabine Steffens:** Conceptualization, Resources, Supervision, Writing – original draft, Writing – review & editing, Funding acquisition. **Stephan Herzig:** Conceptualization, Funding acquisition, Resources, Supervision, Writing – original draft, Writing – review & editing.

## Declaration of competing interest

The authors declare that they have no known competing financial interests or personal relationships that could have appeared to influence the work reported in this paper.

## Data Availability

Data will be made available on request.

## Appendix A Supplementary data

Sup. Figure 1 Phenotypical Changes in Female and Male Mice upon TBL1X /TBL1XR1 Depletion Under HFD/LFD Conditions. A) ipITT, ipGTT results together with changes in glucose, cholesterol and triglycerides in female mice. B) ipITT, ipGTT results together with changes in glucose, cholesterol and triglycerides in male mice. Two-way ANOVA with Tukey's multiple-comparison *post hoc* test. (∗) chow WT vs HFD WT; (^#^) chow KO vs HFD KO. (∗) and (^#^) p < 0.05, (∗∗) and (^##^) p < 0.01. Sup. Figure 2. The depletion of TBL1X/TBL1XR1 in CD4+ T cells changes T Cell profile. A) Reduction in CD3^+^ lymphocytes (as percentage of all lymphocytes) under both chow (WT n = 9, KO n = 7) and HFD (WT n = 13, KO n = 11) conditions. B) Increased CD8^+^ T cell population (% CD4+ of CD3+ cells), (same as in Sup. Figure 2A). C) Reduction in Naïve CD8^+^ T cells and a significant increase in the CD8^+^ central memory (CD44^+^ CD62L^+^) T cell population upon TBL1X/TBL1XR1 depletion in male mice as compared to wild-type littermates, irrespective of the diet, chow (WT n = 3, KO n = 2) and HFD-fed (WT n = 5, KO n = 6). D) CD8^+^ TBL1X/TBL1XR1 KO cells resulted in increased expression of pro-inflammatory mediators, including IFNgamma, TNFalpha (n.s., p = 0,9 and p = 0,3) and IL2, upon ionomycin/PMA-treatment of isolated cells in males, chow (WT n = 3, KO n = 2) and HFD-fed (WT n = 5, KO n = 6). Two-way ANOVA with Tukey's multiple-comparison *post hoc* test, (∗) p < 0.05, (∗∗∗∗) p < 0.0001. Sup. Figure 3. Transcriptomic separation and regulatory signatures in WT and TBL1X/TBL1XR1-deficient CD4^+^ T cells. (A) Principal Component Analysis (PCA) showing clear separation of WT and KO CD4^+^ T cells. (B) Network analysis of differentially expressed genes demonstrating cytokine- and lipid-responsive regulators converging on TBL1X/TBL1XR1. (C) Oxidative-stress–related gene changes, with Srxn1 upregulated and Bach2/Pak1 downregulated in KO cells. Sup. Figure 4. En face aorta lesion quantification of descending aorta. No major changes in plaque development were identified in the descending aortic regions.Descending aorta segments were processed en face only; therefore plaque composition (fibrous cap, necrotic core, collagen, apoptosis) was not evaluated for this region. Sup.Figure 5. Male, female and pooled analyses of T-cell phenotypes. Across all three presentations, the overall patterns are consistent and do not reveal any sex-dependent differences in the proportions or activation states of T-cell subsets. Although minor numerical variation is expected, the direction and magnitude of the responses are comparable between males and females, indicating that sex does not meaningfully influence the T-cell phenotype in this model. Sup. Figure 1
