# Supplementary figures and images for: Nuclear receptor co-factor TBL1X/TBL1XR1 T cell activity protects against atherosclerosis
Source: Mol Metab. 2026 Jan 13;104:102318. doi: 10.1016/j.molmet.2026.102318 (PMC12865552; doi:10.1016/j.molmet.2026.102318)

Sup.Figure 1.

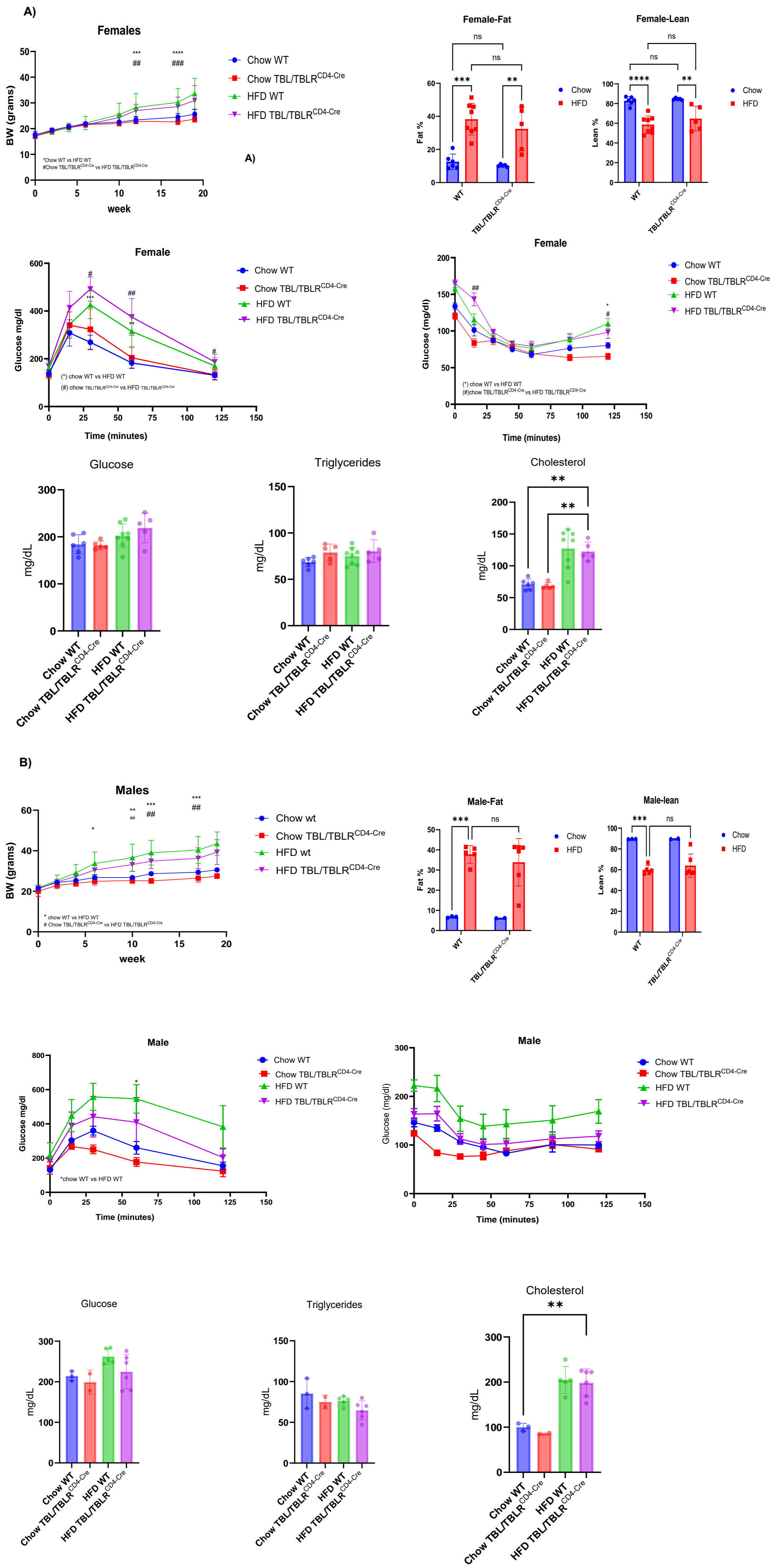

Sup.Figure 2.

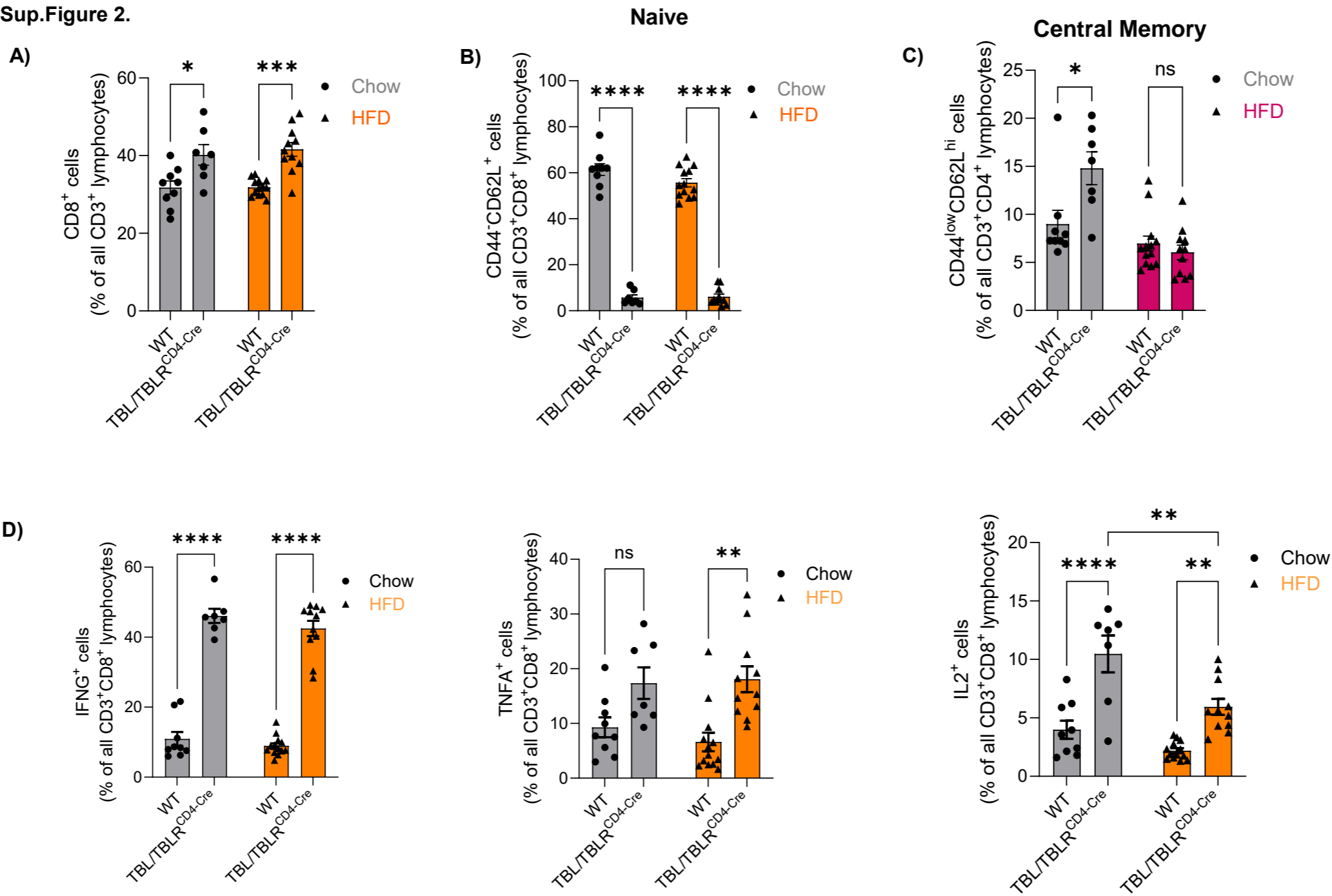

Sup.Figure 3

A)

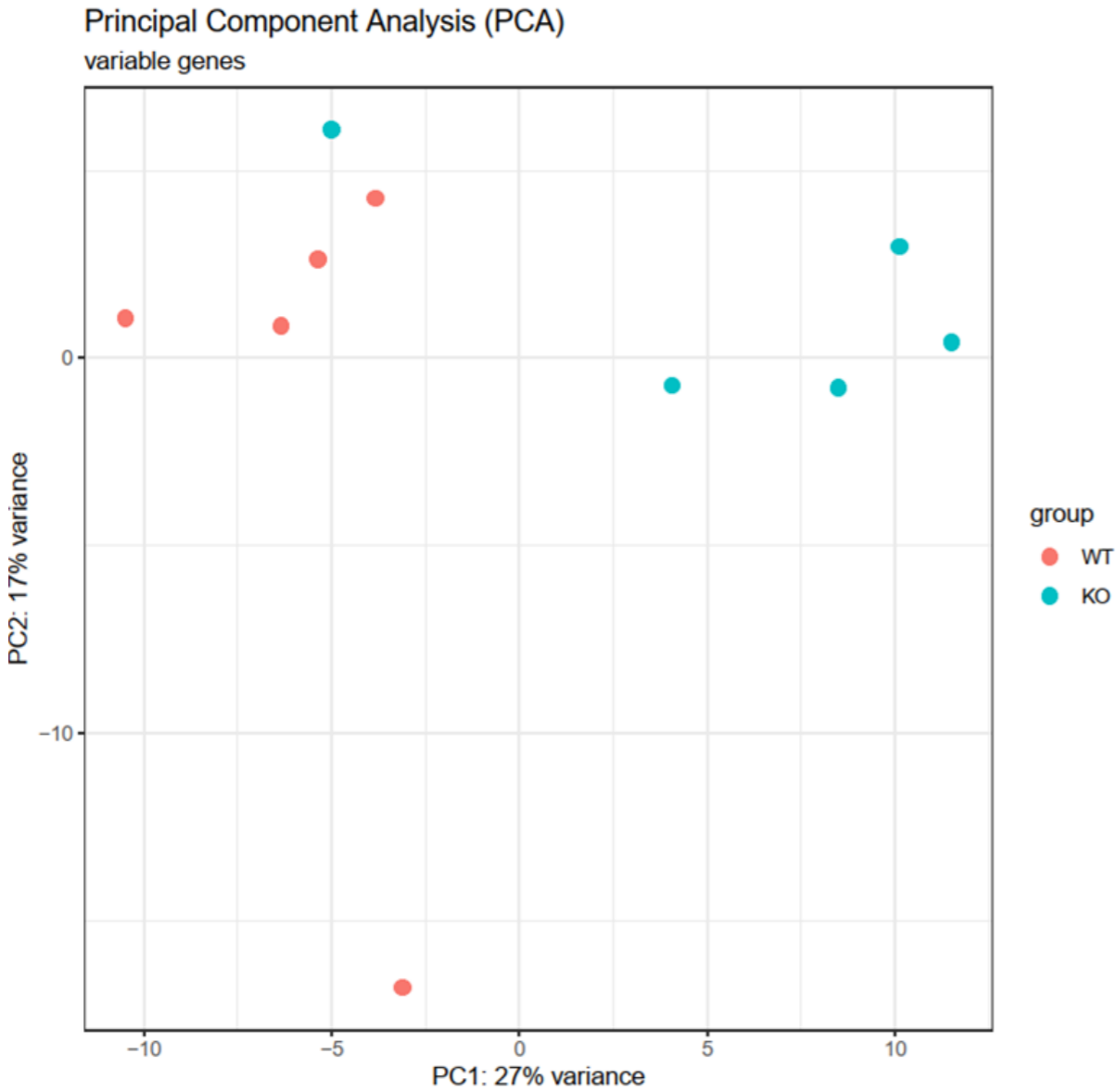

B)

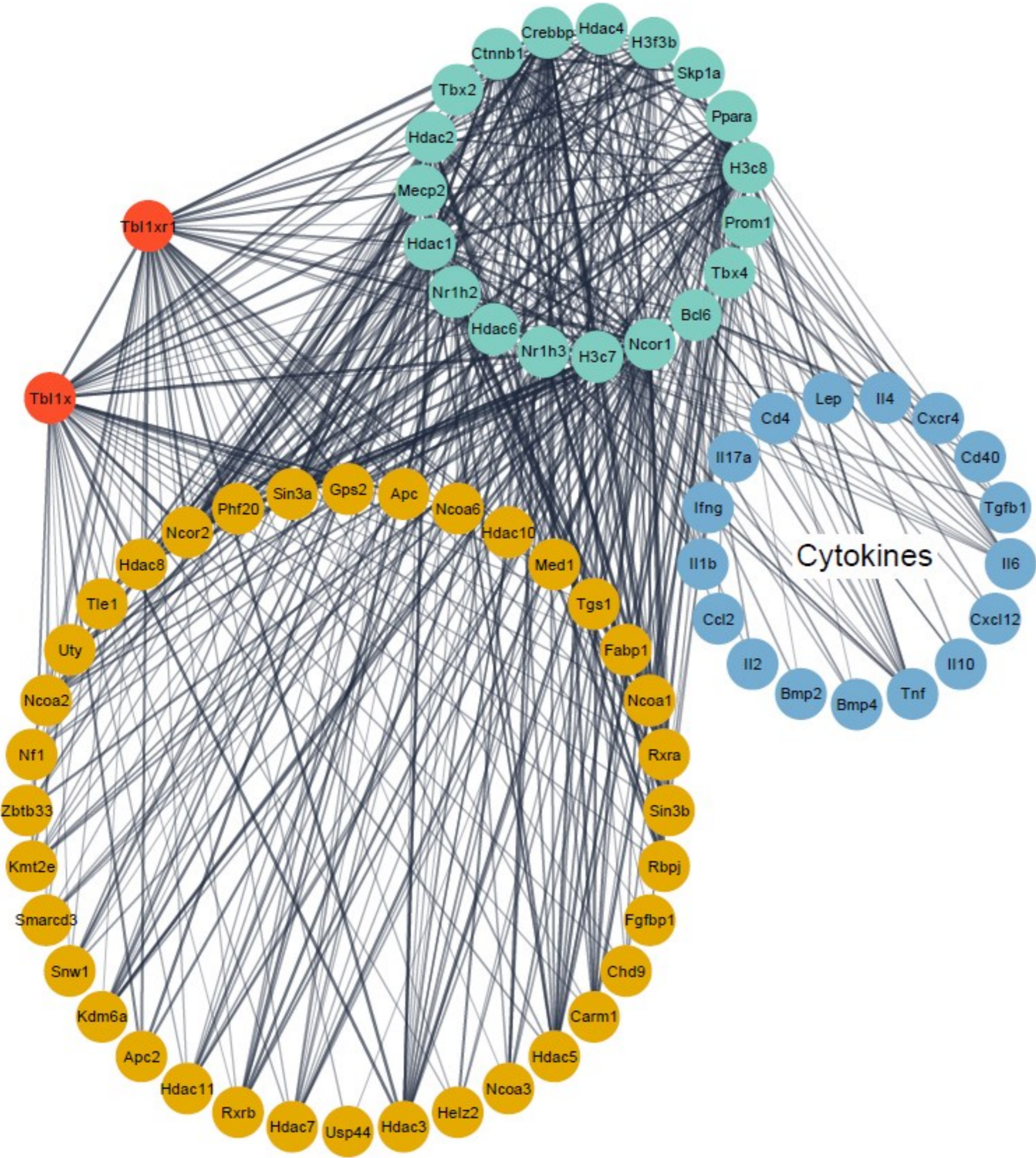

C)

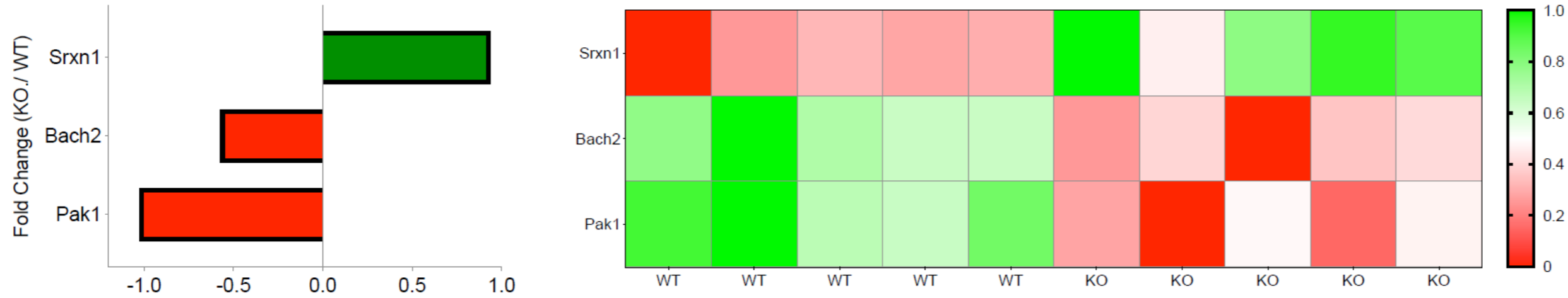

Sup.Figure 4.

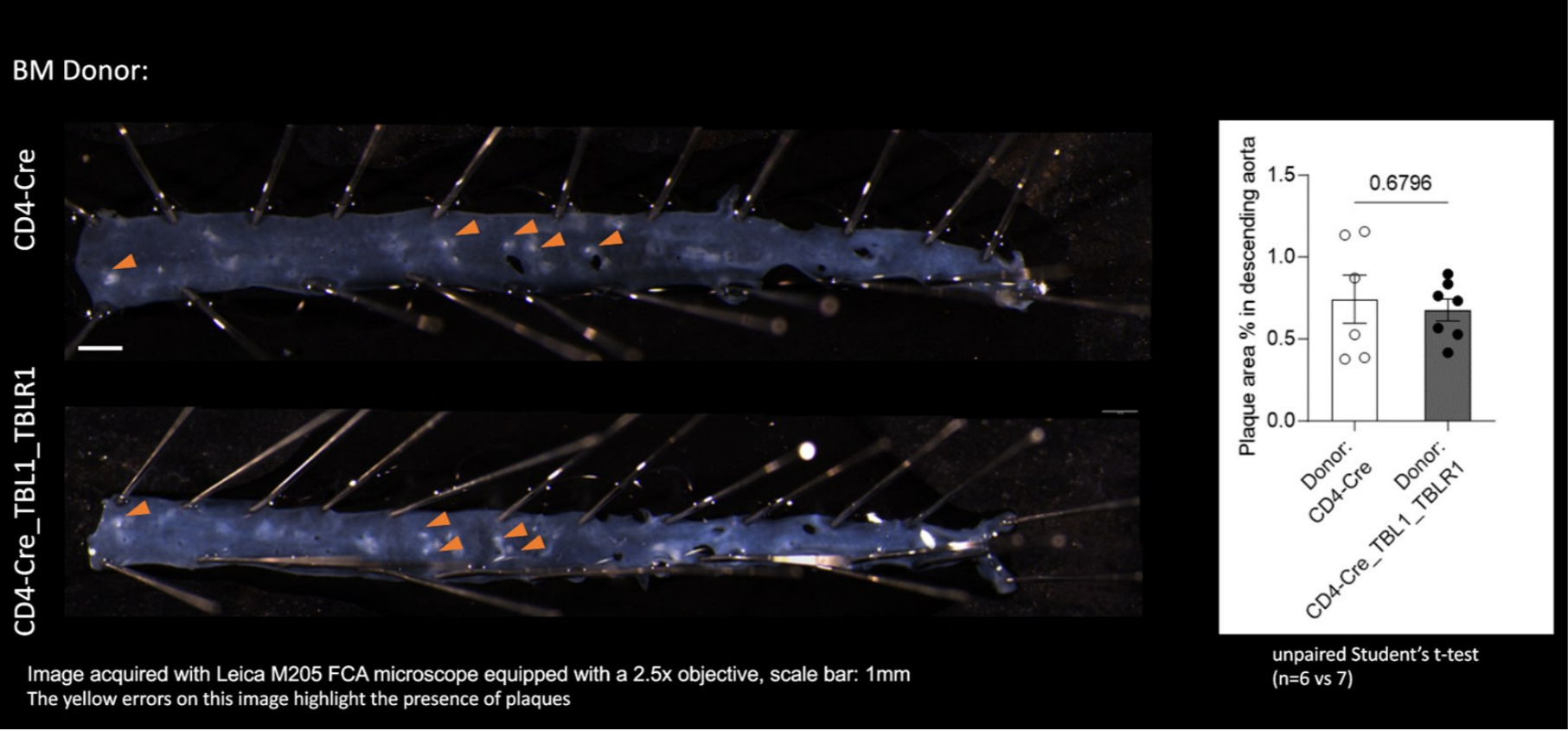

Sup.Figure 5A.

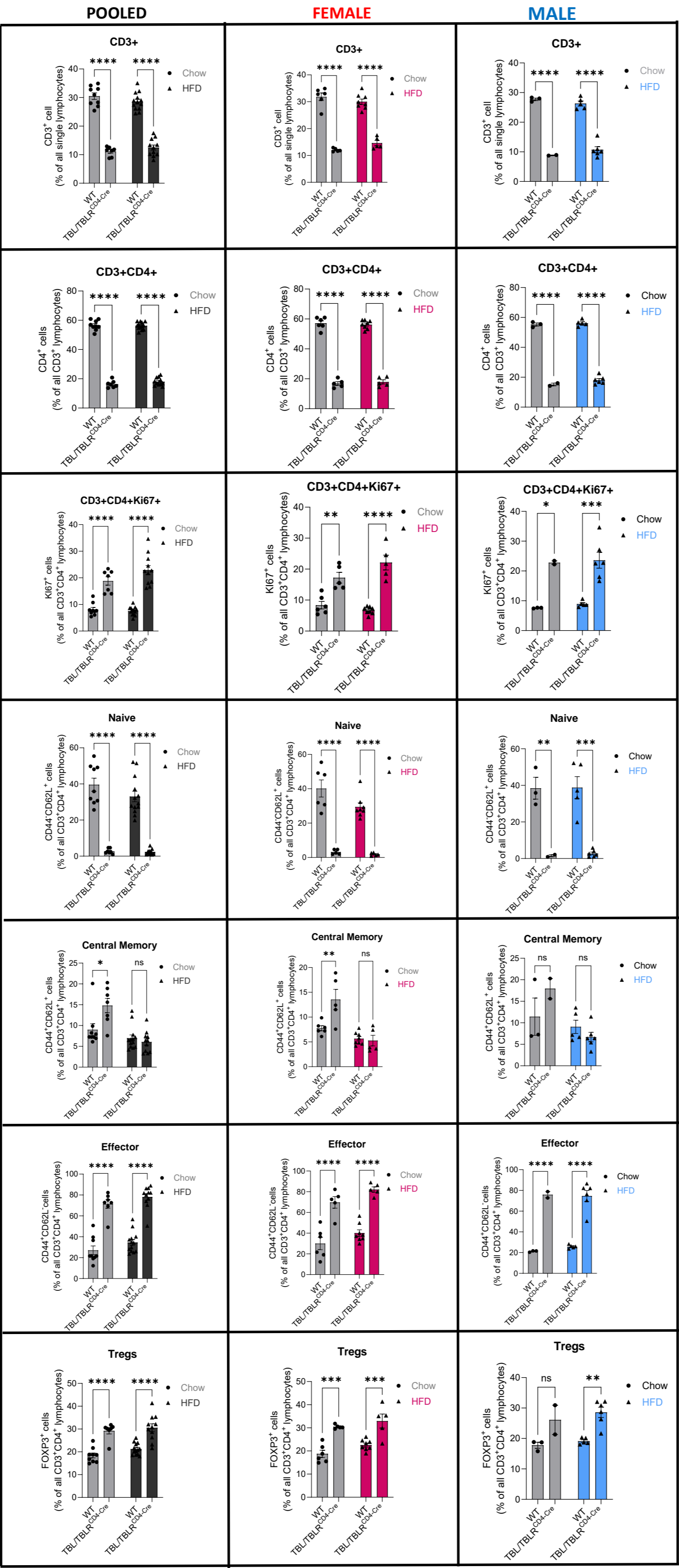

Sup.Figure 5B.

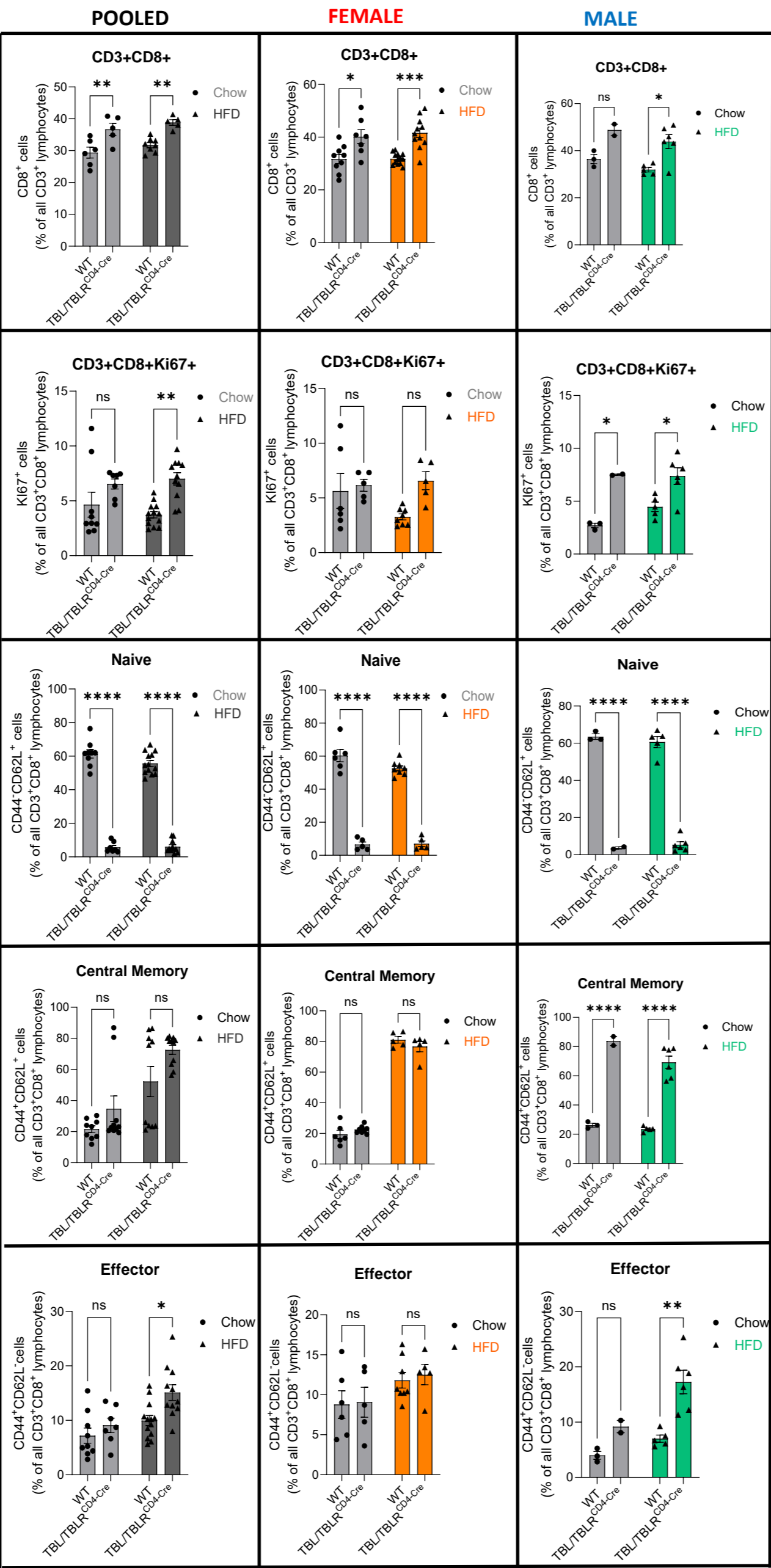

Supplement: Sup. Figure 1 — Phenotypical Changes in Female and Male Mice upon TBL1X /TBL1XR1 Depletion Under HFD/LFD Conditions. A) ipITT, ipGTT results together with changes in glucose, cholesterol and triglycerides in female mice. B) ipITT, ipGTT results together with changes in glucose, cholesterol and triglycerides in male mice. Two-way ANOVA with Tukey's multiple-comparison post hoc test. (∗) chow WT vs HFD WT; (#) chow KO vs HFD KO. (∗) and (#) p < 0.05, (∗∗) and (##) p < 0.01. Sup. Figure 2. The depletion of TBL1X/TBL1XR1 in CD4+ T cells changes T Cell profile. A) Reduction in CD3+ lymphocytes (as percentage of all lymphocytes) under both chow (WT n = 9, KO n = 7) and HFD (WT n = 13, KO n = 11) conditions. B) Increased CD8+ T cell population (% CD4+ of CD3+ cells), (same as in Sup. Figure 2A). C) Reduction in Naïve CD8+ T cells and a significant increase in the CD8+ central memory (CD44+ CD62L+) T cell population upon TBL1X/TBL1XR1 depletion in male mice as compared to wild-type littermates, irrespective of the diet, chow (WT n = 3, KO n = 2) and HFD-fed (WT n = 5, KO n = 6). D) CD8+ TBL1X/TBL1XR1 KO cells resulted in increased expression of pro-inflammatory mediators, including IFNgamma, TNFalpha (n.s., p = 0,9 and p = 0,3) and IL2, upon ionomycin/PMA-treatment of isolated cells in males, chow (WT n = 3, KO n = 2) and HFD-fed (WT n = 5, KO n = 6). Two-way ANOVA with Tukey's multiple-comparison post hoc test, (∗) p < 0.05, (∗∗∗∗) p < 0.0001. Sup. Figure 3. Transcriptomic separation and regulatory signatures in WT and TBL1X/TBL1XR1-deficient CD4+ T cells. (A) Principal Component Analysis (PCA) showing clear separation of WT and KO CD4+ T cells. (B) Network analysis of differentially expressed genes demonstrating cytokine- and lipid-responsive regulators converging on TBL1X/TBL1XR1. (C) Oxidative-stress–related gene changes, with Srxn1 upregulated and Bach2/Pak1 downregulated in KO cells. Sup. Figure 4. En face aorta lesion quantification of descending aorta. No major changes in plaque de [file mmc1.pdf]
